# Supplementary material for: Immunomodulatory Effects of Multivitamin Complexes Containing Agaricus blazei in Patients Undergoing Curative Resection for Non-Small-Cell Lung Cancer: A Randomized, Double-Blind, Placebo-Controlled Multicenter Pilot Trial
Source: Biomedicines. 2025 Dec 26;14(1):53. doi: 10.3390/biomedicines14010053 (PMC12839190; doi:10.3390/biomedicines14010053)
Supplement: Supplementary file 1 [file biomedicines-14-00053-s001.zip › biomedicines-4042525-supplementary.pdf]

Supplementary Material

Supplementary Table S1. Inclusion and exclusion criteria

**Inclusion criteria**
• Adults aged ≥ 19 years.<br>• Patients with histologically confirmed NSCLC at Stage I, II, or IIIA according to TNM staging (8th edition of the International Association for Lung Cancer).<br>• ECOG PS score of 0–2.<br>• No organ failure and suitable for lung resection based on cardiac and pulmonary function tests.<br>• Adequate renal and hepatic function, as defined by specific laboratory parameters.<br>• Ability to comply with study procedures and voluntarily sign informed consent.<br>• Agreement to abstain from other health supplements, multivitamins, mineral complexes, Agaricus mushroom, and related health functional foods during the study period.
**Exclusion criteria**
• Patients with cancers other than NSCLC.<br>• Patients who had undergone surgery for other cancers within the past 5 years, excluding skin cancers other than melanoma and thyroid cancer.<br>• Patients who have received > 5 mg/day of prednisone (or equivalent doses of corticosteroids) within the past month or those expected to require immunosuppressive therapy during the study period.<br>• History of allogeneic stem cell or solid organ transplantation.<br>• Active acute infections or uncontrolled chronic inflammatory lung diseases. However, well-controlled and stable chronic lung diseases may be enrolled at the investigator's discretion.<br>• Autoimmune diseases.<br>• Uncontrolled chronic diseases (e.g., diabetes mellitus, cardiovascular, hepatic, or renal disorders) or other systemic conditions that could affect immune function.<br>• Psychiatric disorders, substance abuse, or alcohol addiction that may interfere with the study.<br>• Patients deemed unsuitable by the investigator.<br>• Women of childbearing potential.<br>• Pregnant or breastfeeding women.<br>• Illiterate patients.

NSCLC, non-small-cell lung cancer; ECOG PS, Eastern Cooperative Oncology Group Performance Status

Supplementary Table S2. Composition of the investigational product

Ingredient	Amount per capsule
Agaricus blazei powder	1200 mg
Vitamin E	150 mg
Vitamin C	100 mg
Niacin	60 mg
Vitamin B1	25 mg
Vitamin B2	25 mg
Vitamin B6	20 mg
Zinc	8 mg
Manganese	2 mg
Copper	500 µg
Folic Acid	400 µg
Vitamin D	25 µg
Selenium	18 µg
Vitamin B12	6 µg

Supplementary Table S3. Baseline characteristics of the overall population

Variables	Overall (n = 66)	Treatment group (n = 32)	Control group (n = 34)	*p*-value
Age (median, range)	64 (34, 84)	62 (34, 84)	64 (49, 78)	0.594
Female sex	28 (42.4)	14 (43.8)	14 (41.2)	0.833
Smoking history				0.260
Never	31 (47.0)	16 (50.0)	15 (44.1)	
Ex-smoker	16 (24.2)	5 (15.6)	11 (32.4)	
Current smoker	19 (28.8)	11 (34.4)	8 (23.5)	
BMI (kg/m^2)	24.4±2.8	24.1±2.7	24.8±2.9	0.296
Histologic subtype				0.597
Adenocarcinoma	56 (84.8)	26 (81.3)	30 (88.2)	
Squamous cell carcinoma	9 (13.6)	5 (15.6)	4 (11.8)	
Adenosquamous carcinoma	1 (1.5)	1 (3.1)	0	
Pathologic stage				0.380
IA	30 (45.5)	13 (40.6)	17 (50.0)	
IB	15 (22.7)	8 (25.0)	7 (20.6)	
IIB	8 (12.1)	5 (15.6)	3 (8.8)	
IIIA	10 (15.2)	3 (9.4)	7 (20.6)	
IIIB	2 (3.0)	2 (6.3)	0	

IVA	1 (1.5)	1 (3.1)	0	
Type of surgery				0.869
Lobectomy	55 (83.3)	26 (81.3)	29 (85.3)	
Segmentectomy	6 (9.1)	3 (9.4)	3 (8.8)	
Wedge resection	3 (4.5)	2 (6.3)	1 (2.9)	
Sleeve lobectomy	2 (3.0)	1 (3.1)	1 (2.9)	
ECOG PS				0.339
0	27 (40.9)	15 (46.9)	12 (35.3)	
1	39 (59.1)	17 (53.1)	22 (64.7)	
EORTC QLQ-LC29 score	32.1±5.2	32.1±4.8	32.1±5.5	0.995

BMI, body mass index; ECOG PS, Eastern Cooperative Oncology Group Performance Status; EORTC QLQ-LC29, European Organization for Research and Treatment of Cancer-Quality of Life Questionnaire-Lung Cancer 29

*Data are presented as number (%) or mean ± standard deviation.

Supplementary Table S4. Distribution of responses to the five symptom-related items in the EORTC QLQ-LC29 at V3

Questionnaire items	**Overall**<br>**(n = 53)**	**Treatment group (n = 29)**	**Control group**<br>**(n = 24)**	***p*-value**
Pain at the surgical site				0.896
Not at all	13 (24.5)	7 (24.1)	6 (25.0)	
A little	28 (52.8)	14 (48.3)	14 (58.3)	
Quite a bit	4 (7.5)	2 (7.0)	2 (8.3)	
Very much	4 (7.5)	3 (10.3)	1 (4.2)	
Hypersensitivity at the surgical site				>0.999
Not at all	48 (90.6)	25 (86.2)	23 (95.8)	
A little	1 (1.9)	1 (3.4)	0	
Physical activity limitations after surgery				0.169
Not at all	16 (30.2)	8 (27.6)	8 (33.3)	
A little	22 (41.5)	12 (41.4)	10 (41.7)	
Quite a bit	7 (13.2)	2 (6.9)	5 (20.8)	
Very much	4 (7.5)	4 (13.8)	0	
Difficulty with arm or shoulder motion				0.714
Not at all	36 (67.9)	17 (58.6)	19 (79.2)	
A little	14 (26.4)	8 (27.6)	6 (25.0)	

Quite a bit	3 (5.7)	1 (3.4)	2 (8.3)	
Very much	1 (1.9)	1 (3.4)	0	
Discomfort in daily life after surgery				0.648
Not at all	26 (49.1)	14 (48.3)	12 (50.0)	
A little	19 (35.8)	11 (37.9)	8 (33.3)	
Quite a bit	2 (3.8)	0	2 (8.3)	
Very much	2 (3.8)	1 (3.4)	1 (4.2)	

Data are presented as number (%).

(A)

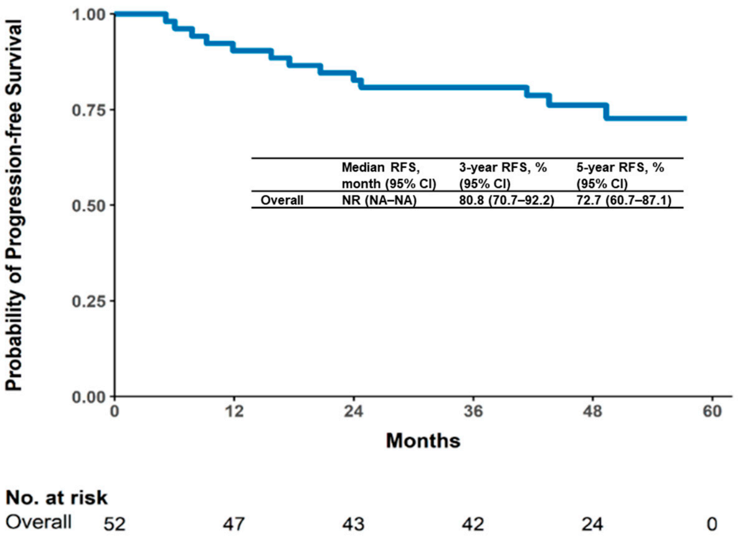

(B)

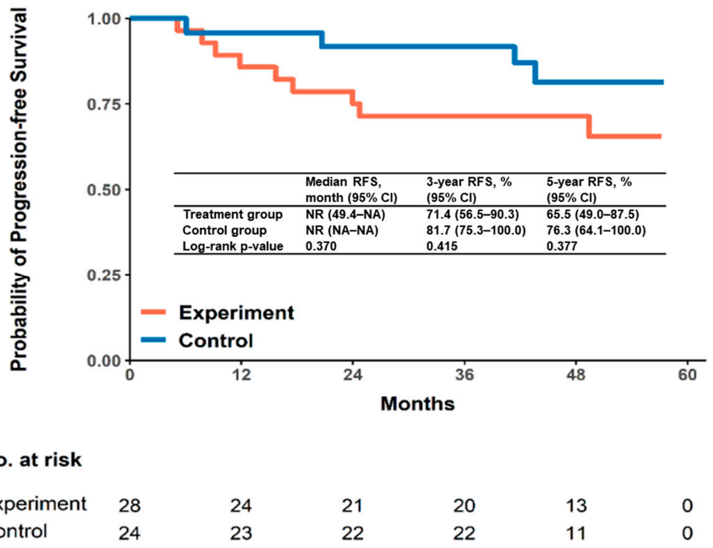

Supplementary Figure S1. Progression-free survival of the study population (A) and treatment group (B)
